# 22q11 Deletion Syndrome and Urogenital Manifestations: A Clinicopathological Case Report

**DOI:** 10.3389/fmed.2016.00053

**Published:** 2016-11-28

**Authors:** M. Vachette, G. E. Grant, J. Bouquet de la Joliniere, M. Jotterand, N. Ben Ali, A. Feki, R. Capoccia Brugger

**Affiliations:** ^1^Department of Gynecology and Obstetrics, HFR, Fribourg, Switzerland; ^2^Institute of Pathology, CHUV, Lausanne, Switzerland; ^3^Department of Gynecology and Obstetrics, HNE, Neuchâtel, Switzerland

**Keywords:** 22q11 deletion syndrome, DiGeorge syndrome, CATCH22 syndrome, urogenital manifestations, renal agenesis, hypospadias

## Abstract

**Background:**

Deletion in the chromosomal region 22q11 results from the abnormal development of the third and fourth pharyngeal pouches during embryonic life and presents an expansive phenotype with more than 180 clinical features described that involve every organ and system.

**History and signs:**

A 23-year-old African woman presented for the first trimester echography, which revealed an isolated anechoic structure suggesting a ureteral dilatation. The suspicion of a malposition of great arteries in the second trimester indicated an amniocentesis leading to a diagnosis of 22q11 deletion.

**Outcome:**

At 32 weeks, the patient was admitted for premature rupture of membranes and gave birth 2 weeks later to a male newborn who presented a respiratory distress syndrome and probably died secondary to a tracheal stenosis. Necropsy revealed typical clinical features of 22q11 deletion associated with left renal agenesis, hypospadias, and penile hypoplasia.

**Conclusion:**

We report a case of 22q11 deletion syndrome with typical clinical features associated with urogenital manifestations suspected at the first trimester ultrasound.

## Introduction

**Figure 1:**
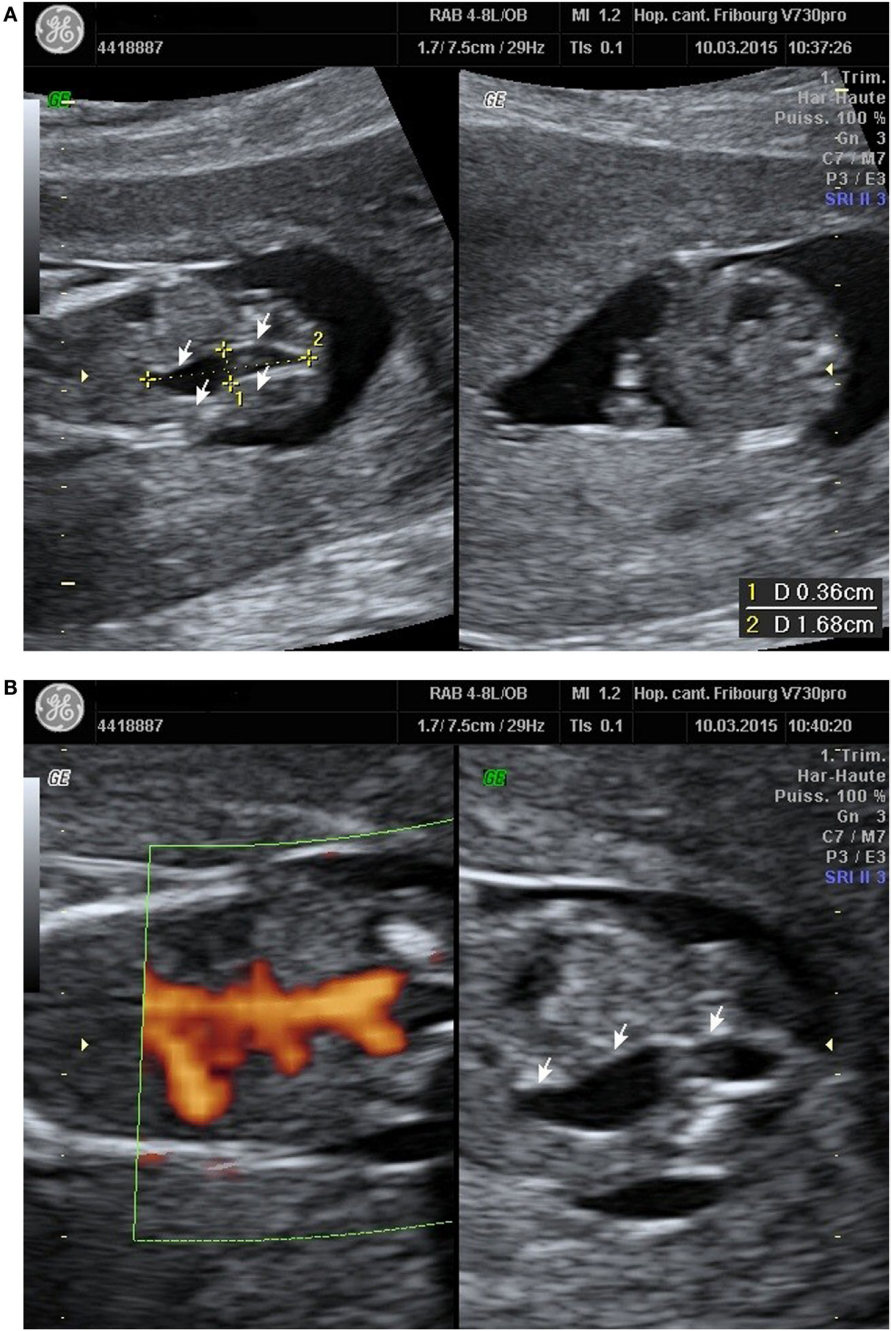
**(A)** Right ureteral dilatation. **(B)** Doppler renal arteries.

A 23-year-old healthy African woman, mother of a healthy child, born by C-section for obstructed labor, presented at 12 weeks and 5 days’ gestation of the second spontaneous pregnancy for the first trimester echography. The exam revealed an anechoic structure appearing like a possible right ureteral dilatation (Figure 1A). Both renal arteries seemed to be present with the Doppler evaluation (Figure 1B). First trimester scan exam including nuchal translucency was otherwise normal and combined test for Down syndrome has been evaluated at 1/30,000.

At 18 weeks’ gestation, we had a strong suspicion of great arteries’ malposition because of an abnormal three-vessel view. Regarding this suspicion, an amniocentesis was performed and revealed a male fetus with 22q11 deletion. The follow-up was made in a university center, and several echographic exams were performed. The first one at 19 week’s gestation concluded to an anomalous pulmonary venous return and transposition of great vessels. The left renal agenesis was discovered at 25 weeks’ gestation, and the ultrasound at 29 weeks’ gestation showed a conotruncal cardiopathy with malposition of great arteries, interventricular communication, left renal agenesis, and penile hypoplasia. The transposition of great vessels described above was nullified.

**Figure 2:**
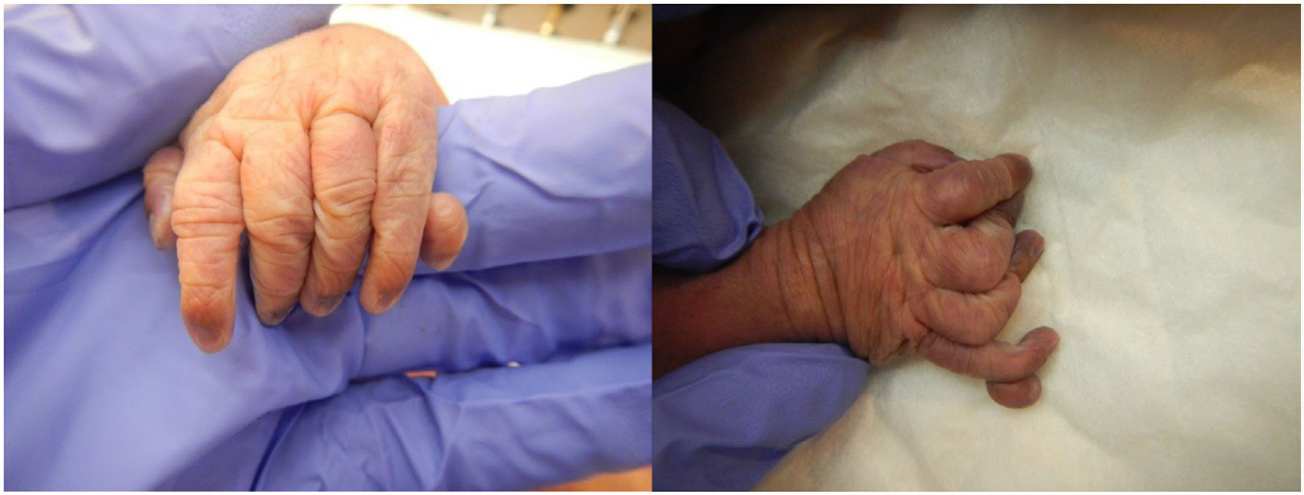
**Postaxial bilateral hexadactyly**.

**Figure 3:**
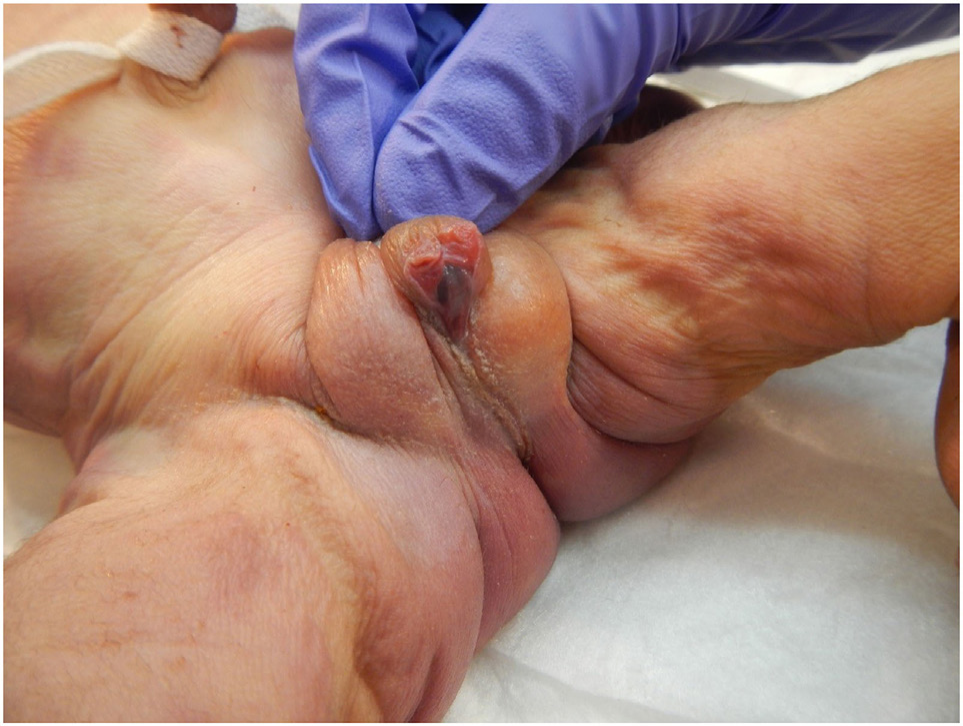
**Penile hypoplasia and perineoscrotal hypospadias**.

**Figure 4:**
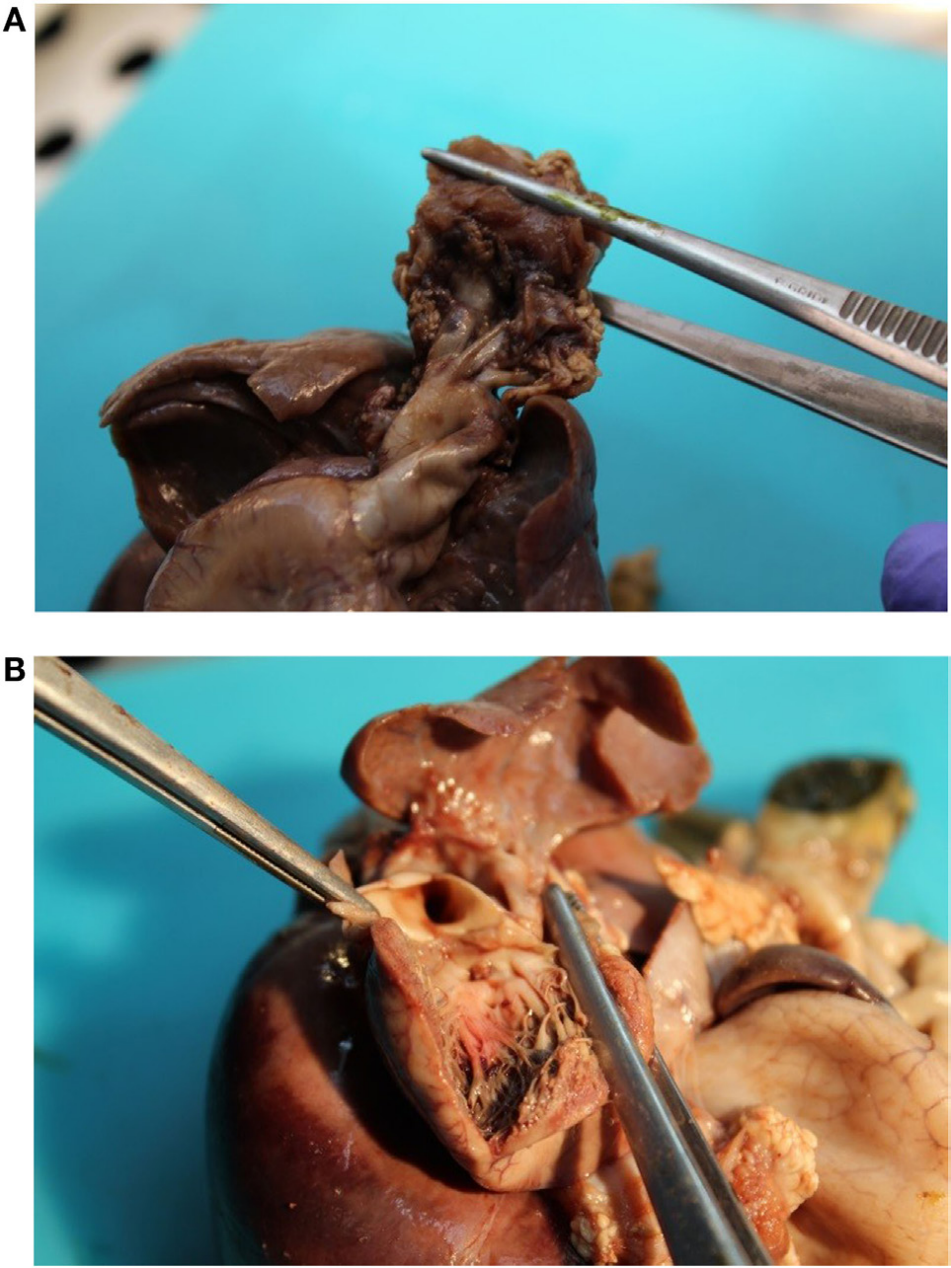
**(A)** Malposition of the aortic orifice and s shape of the ascending aorta without transposition of great vessels. **(B)** Two punctate interventricular communications.

The patient presented for premature rupture of membrane at 32 weekends and 4 days’ gestation and delivered 2 weeks later a male fetus of 1578 g with the following features at necropsy: facial anomalies with hypotelorism, wide nasal bridge, low set right ear and microcephaly, and limb manifestations with postaxial bilateral hexadactyly without bone component (Figure 2). About urogenital anomalies, left renal agenesis and perineoscrotal hypospadias with penile hypoplasia (Figure 3) with descended testes were found. Concerning the cardiovascular and respiratory system, the exam revealed pneumothorax with subglottic perforation and tracheal stenosis, parathyroid aplasia, heart defect with “s shape” of ascending aorta without transposition (Figure 4A), and two punctate interventricular communications, one muscular and the other one, sub-arterial (Figure 4B). The baby probably died secondary to a tracheal stenosis, which limited the intubation.

## Background

First described in 1968, deletions in the chromosomal region 22q11 result from an anomaly of the third and fourth pharyngeal pouches during embryonic life (1). Also known as DiGeorge syndrome, velocardiofacial syndrome, and CATCH22 acronym (cardiac defects, abnormal faces, thymus hypoplasia, clef palate, and hypocalcemia) that outlines the main clinical features (2), this deletion presents an expansive phenotype with more than 180 clinical features described that involve every organ and system (3). It occurs in 1/4000 live births and constitutes the most frequent interstitial chromosomal aberration (4).

Researchers are working to identify all of the genes that contribute to the features of 22q11.2 deletion syndrome (5). They have determined that the loss of a particular gene on chromosome 22, *TBX1*, is probably responsible for many of the syndrome’s characteristic signs (such as heart defects, a cleft palate, distinctive facial features, hearing loss, and low calcium levels). The loss of additional genes in the deleted region likely contributes to the varied features of 22q11.2 deletion syndrome (6).

## Discussion

The original description of the syndrome was derived from a published discussion at an immunology meeting in 1965. DiGeorge published a formal report 3 years later.

Since then, many terms have been proposed to describe this syndrome. It seems appropriate to use Takao syndrome for cases with a preponderant cardiac presentation in contrast to the low T cells and hypocalcemic presentation in infancy of DiGeorge syndrome and the craniofacial and palatal abnormalities typical of Shprintzen syndrome or velocardiofacial syndrome. In 1993, Wilson et al. proposed to see DiGeorge syndrome as the severe end of the clinical spectrum embraced by the acronym CATCH22 syndrome (7).

The acronym CATCH22 includes the classical manifestations as cardiac abnormality, facial abnormality, thymus hypoplasia, cleft palate, and hypocalcemia due to hypoparathyroidism.

However, in our case, we would like to focus on urogenital manifestations, whereas several authors described features in every organ and system.

The etiology of renal malformations in deletion 22q11 still remains unclear, but it is postulated that a single-gene defect within the DGCR is responsible for the development of the urogenital tract in early embryonic development (4). Described renal malformations in patients with 22q11 deletion syndrome are absent, dysplastic or multicystic kidneys, obstructive abnormalities, vesicoureteral reflux, nephron calcinosis, duplex kidney, and affect 10–36% of patients according to the different authors (7–9). Concerning genital anomalies, which are less frequent, it has been reported cases of absent uterus, uterine didelphys, undescended testes, small penis, hypospadias, and shawl scrotum (8–11).

Other possible anechoic structures (ovarian cyst, intestinal duplication, and mesenteric cyst) usually appear in the second trimester. In our case, the sonographic anomaly was not present at the 18-week ultrasound.

CATCH22 syndrome is usually sporadic and results from *de novo* deletion within chromosome 22 without major difference between parents of origin (8). However, inherited transmission has been reported in 6–28% of patients (12), emphasizing the need for studying both parents when a child is found to have a deletion (8), the risk of a further pregnancy with monosomy 22q11 for these couples being obviously 50% (7).

Even the largest of the published series are still too small to provide reliable data for counseling (8), it seems reasonable to offer to patients with 22q11 deletion, prenatal detection by choriocentesis or amniocentesis (13). Concerning normal parents of offspring with a *de novo* deletion, the recurrence in subsequent pregnancies is probably low, but a germline mosaicism for a 22q11 deletion has not been excluded (13). Furthermore, cytogenetic and molecular evaluation of the fetus for a 22q11 deletion should be systematically offered to the parents when a conotruncal heart malformation is detected prenatally. With the advent of preimplantation diagnosis, it is important to know that it is now feasible to detect CATCH22 at the blastocyst stage, making the preimplantation diagnosis possible.

## Concluding Remarks

As described earlier, kidney malformations in the 22q11 syndrome are not so rare. Despite this, our case is distinguished by the discovery of prenatal abnormalities and the presence of several genital defects.

Although urogenital malformations are not the main sign for CATCH22 syndrome, it is really important to always perform a good morphological exam and given their prevalence in this syndrome, if this latter is suspected, to study precisely urinary and genital tract.

Many of these abnormalities require surgical or medical intervention to prevent complications, such as hypertension and renal nephropathy (8) reason, why early diagnosis and treatment is recommended.

## Author Contributions

All authors listed have made substantial, direct, and intellectual contribution to the work and approved it for publication.

## Conflict of Interest Statement

The authors declare that the research was conducted in the absence of any commercial or financial relationships that could be construed as a potential conflict of interest.
